# Supplementary material for: Reduction of knee joint load suppresses cartilage degeneration, osteophyte formation, and synovitis in early-stage osteoarthritis using a post-traumatic rat model
Source: PLoS One. 2021 Jul 16;16(7):e0254383. doi: 10.1371/journal.pone.0254383 (PMC8284605; doi:10.1371/journal.pone.0254383)
Supplement: S1 Table — (DOCX) [file pone.0254383.s001.docx]

**S1 Table. Subchondral bone damage score**

| Grade | Description |
| --- | --- |
| 0 | No changes |
| 1 | Increased basophilia at tidemark  No fragmentation of tidemark  No marrow changes or, if present, minimal and focal  Increased thickening of subchondral bone subjacent to the area of greatest articular cartilage lesion severity |
| 2 | Increased basophilia at tidemark  Minimal to mild focal fragmentation of calcified cartilage of tidemark  Mesenchymal change in marrow (fibroblastic cells) involving about 1/4 of subchondral region under lesion  Increased thickening of subchondral bone subjacent to the area of greatest articular cartilage lesion severity. |
| 3 | Increased basophilia at tidemark  Mild to marked fragmentation (multiple larger areas) of calcified cartilage/subchondral bone loss Mesenchymal change in marrow in up to 3/4 of total area  Areas of marrow chondrogenesis may be evident but no major collapse of articular cartilage into epiphyseal bone (definite depression in surface) |
| 4 | Increased basophilia at tidemark  Marked to severe fragmentation of calcified cartilage  Marrow mesenchymal change involves up to 3/4 of area  Articular cartilage has collapsed into the epiphysis to a depth of 250 mm or less from tidemark (see definite depression in surface cartilage). |
| 5 | Increased basophilia at tidemark  Marked to severe fragmentation of calcified cartilage  Marrow mesenchymal change involves up to 3/4 of area  Articular cartilage has collapsed into the epiphysis to a depth of greater than 250 mm from tidemark. |
