# Supplementary material for: Reduction of knee joint load suppresses cartilage degeneration, osteophyte formation, and synovitis in early-stage osteoarthritis using a post-traumatic rat model
Source: PLoS One. 2021 Jul 16;16(7):e0254383. doi: 10.1371/journal.pone.0254383 (PMC8284605; doi:10.1371/journal.pone.0254383)
Supplement: S2 Table — (DOCX) [file pone.0254383.s002.docx]

**S2 Table. Scoring system for synovitis**

| Criteria | Score | Morphological feature |
| --- | --- | --- |
| Hyperplasia / enlargement of synovial lining cell layer | 0 | Absent |
|  | 1 | Slight |
|  | 2 | Moderate |
|  | 3 | Strong |
| Inflammatory infiltration | 0 | Absent |
|  | 1 | Slight |
|  | 2 | Moderate |
|  | 3 | Strong |
| Activation of synovial stroma / pannus formation | 0 | Absent |
|  | 1 | Slight |
|  | 2 | Moderate |
|  | 3 | Strong |
