# Supplementary material for: Reduction of knee joint load suppresses cartilage degeneration, osteophyte formation, and synovitis in early-stage osteoarthritis using a post-traumatic rat model
Source: PLoS One. 2021 Jul 16;16(7):e0254383. doi: 10.1371/journal.pone.0254383 (PMC8284605; doi:10.1371/journal.pone.0254383)
Supplement: S3 Table — (DOCX) [file pone.0254383.s003.docx]

**S3 Table. Changes in the body weights (g) of the rats throughout the experiment.**

**At 2 weeks**

|  | OA group | OAHS group |
| --- | --- | --- |
| At DMM | 421.0 ± 14.3  (403.1–438.8) | 428.0 ± 27.8  (393.4–462.5) |
| At 2 weeks | 431.6 ± 17.3  (410.1–453.0) | 395.4 ± 24.2*  (365.3–425.4) |

**At 4 weeks**

|  | OA group | OAHS group |
| --- | --- | --- |
| At DMM | 403.4 ± 32.7  (362.7–444.0) | 410.4 ± 35.7  (366.0–454.7) |
| At 4 weeks | 429.0 ± 21.2  (402.5–455.4) | 432.2 ± 27.7  (397.6–466.7) |

Mean ± SD (95% CI)

* The result was significantly different from those of OA group at the same time.

*P* < .05 for all.
