# Supplementary material for: Reduction of knee joint load suppresses cartilage degeneration, osteophyte formation, and synovitis in early-stage osteoarthritis using a post-traumatic rat model
Source: PLoS One. 2021 Jul 16;16(7):e0254383. doi: 10.1371/journal.pone.0254383 (PMC8284605; doi:10.1371/journal.pone.0254383)
Supplement: S4 Table — (DOCX) [file pone.0254383.s004.docx]

**S4 Table. Histological scores**

**Maximum OARSI score**

|  | OA group | | OAHS group | |
| --- | --- | --- | --- | --- |
|  | Operated | Sham | Operated | Sham |
| At 2 weeks | 4 (3–7.5) | 0 (0–0) | 1 (1–3) * | 0 (0–0) |
| At 4 weeks | 8 (6–9) | 0 (0–0) | 3 (2–4) * | 0 (0–0) |

**Summed OARSI score**

|  | OA group | | OAHS group | |
| --- | --- | --- | --- | --- |
|  | Operated | Sham | Operated | Sham |
| At 2 weeks | 8 (5.5–11) | 0 (0–0) | 1 (1–3) * | 0 (0–0) |
| At 4 weeks | 17 (15.5–19) | 0 (0–0) | 4 (3.5–5.5) * | 0 (0–0) |

**Subchondral bone damage score**

|  | OA group | | OAHS group | |
| --- | --- | --- | --- | --- |
|  | Operated | Sham | Operated | Sham |
| At 2 weeks | 0 (0–0) | 0 (0–0) | 0 (0–0) | 0 (0–0) |
| At 4 weeks | 0 (0–1) | 0 (0–0) | 0 (0–0) | 0 (0–0) |

**Synovial inflammation score**

|  | OA group | | OAHS group | |
| --- | --- | --- | --- | --- |
|  | Operated | Sham | Operated | Sham |
| At 2 weeks | 3 (2.5–3) | 1 (0–2) | 2 (2–3) | 0 (0–1) |
| At 4 weeks | 1 (1–4) | 0 (0–0) | 2 (2–2.5) | 0 (0–0) |

Median (lower quartile–upper quartile)

* The result was significantly different from those of OA group at the same time.

*P* < .05 for all.
