# Supplementary material for: Reduction of knee joint load suppresses cartilage degeneration, osteophyte formation, and synovitis in early-stage osteoarthritis using a post-traumatic rat model
Source: PLoS One. 2021 Jul 16;16(7):e0254383. doi: 10.1371/journal.pone.0254383 (PMC8284605; doi:10.1371/journal.pone.0254383)
Supplement: S5 Table — (DOCX) [file pone.0254383.s005.docx]

**S5 Table. Histomorphometrical results**

**Cartilage thickness (µm)**

|  | OA group | | OAHS group | |
| --- | --- | --- | --- | --- |
|  | Operated | Sham | Operated | Sham |
| At 2 weeks | 332.7 ± 27.2  (298.9–366.5) | 428.0 ± 30.0**  (390.7–465.4) | 328.8 ± 15.4  (309.6–348.0) | 424.7 ± 15.6**  (405.2–444.1) |
| At 4 weeks | 253.2 ± 39.7  (203.9–302.6) | 391.1 ± 33.3**  (349.7–432.5) | 304.6 ± 31.2  (265.8–343.4) | 413.5 ± 15.7**  (393.9–433.1) |

**Matrix intensity (pixel value)**

|  | OA group | | OAHS group | |
| --- | --- | --- | --- | --- |
|  | Operated | Sham | Operated | Sham |
| At 2 weeks | 44.5 ± 17.6  (22.6–66.4) | 40.3 ± 9.0  (29.1–51.5) | 45.2 ± 5.3  (38.5–51.8) | 39.1 ± 3.9  (34.2–43.9) |
| At 4 weeks | 71.1 ± 25.6  (39.3–103.0) | 41.1 ± 10.5**  (28.0–54.3) | 48.6 ± 15.4  (29.4–67.8) | 50.0 ± 7.7  (40.4–59.6) |

**Chondrocyte density (cells/mm^2^)**

|  | OA group | | OAHS group | |
| --- | --- | --- | --- | --- |
|  | Operated | Sham | Operated | Sham |
| At 2 weeks | 869.2 ± 92.4  (754.4–984.1) | 1150.6 ± 186.1**  (919.5–1381.7) | 1197.8 ± 189.7*  (962.2–1433.4) | 1130.7 ± 139.7  (1081.3–1180.0) |
| At 4 weeks | 679.5 ± 160.8  (479.8–879.2) | 1016.6 ± 98.8**  (893.9–1139.4) | 1177.6 ± 173.1*  (962.6–1392.5) | 1062.5 ± 132.8  (897.6–1227.4) |

**Osteophyte length (µm)**

|  | OA group | | OAHS group | |
| --- | --- | --- | --- | --- |
|  | Operated | Sham | Operated | Sham |
| At 2 weeks | 317.3 ± 76.8  (221.8–412.7) | 181.5 ± 13.9**  (164.1–198.8) | 251.0 ± 24.5  (220.5–281.4) | 160.3 ± 11.7**  (145.7–175.0) |
| At 4 weeks | 350.6 ± 55.4  (281.8–419.4) | 201.8 ± 20.9**  (175.8–227.9) | 260.9 ± 13.8*  (243.7–278.2) | 174.2 ± 27.0**  (140.5–207.8) |

**Synovial thickness (µm)**

|  | OA group | | OAHS group | |
| --- | --- | --- | --- | --- |
|  | Operated | Sham | Operated | Sham |
| At 2 weeks | 8.8 ± 1.1  (7.4–10.3) | 8.1 ± 1.4  (6.3–9.9) | 5.7 ± 0.6*  (4.8–6.5) | 5.5 ± 1.0  (4.3–6.8) |
| At 4 weeks | 7.0 ± 1.6  (4.9–9.2) | 4.1 ± 0.7**  (3.2–5.1) | 6.7 ± 1.2  (5.2–8.2) | 4.2 ± 0.3**  (3.9–4.6) |

Mean ± SD (95% CI), *P* < .05 for all.

* * The result was significantly different from those of OA group at the same time.

** * The result was significantly different from operated limb of the same group at the same time.
